# Artificial intelligence for improving decision-making in bacterial infection management: a narrative review

**DOI:** 10.1093/jac/dkaf470

**Published:** 2025-12-22

**Authors:** Anisia Talianu, Oskar Fraser-Krauss, William Bolton, Damien Ming, Nina Zhu, Bernard Hernandez, Mark Gilchrist, Alison Holmes, Pantelis Georgiou, Timothy Miles Rawson

**Affiliations:** Faculty of Engineering, Department of Computing, Imperial College London, London, UK; Centres for Antimicrobial Optimisation Network, Imperial College London, London, UK; Faculty of Engineering, Department of Computing, Imperial College London, London, UK; Centres for Antimicrobial Optimisation Network, Imperial College London, London, UK; Faculty of Engineering, Department of Computing, Imperial College London, London, UK; Centres for Antimicrobial Optimisation Network, Imperial College London, London, UK; Centres for Antimicrobial Optimisation Network, Imperial College London, London, UK; The Fleming Initiative, Imperial College London, London, UK; Department of Infectious Diseases, Imperial College Healthcare NHS Trust, London, London, UK; Centres for Antimicrobial Optimisation Network, Imperial College London, London, UK; The Fleming Initiative, Imperial College London, London, UK; Centres for Antimicrobial Optimisation Network, Imperial College London, London, UK; The Fleming Initiative, Imperial College London, London, UK; Department of Infectious Diseases, Imperial College Healthcare NHS Trust, London, London, UK; Centres for Antimicrobial Optimisation Network, Imperial College London, London, UK; The Fleming Initiative, Imperial College London, London, UK; David Price Evans Infectious Diseases & Global Health Group, University of Liverpool Faculty of Medicine, Liverpool, UK; Department of Infectious Disease, Imperial College London, London, UK; Faculty of Engineering, Department of Electrical and Electronic Engineering, Imperial College London, London, UK; Centre for Bio-Inspired Technology, Imperial College London, London, UK; Centres for Antimicrobial Optimisation Network, Imperial College London, London, UK; The Fleming Initiative, Imperial College London, London, UK; Department of Infectious Diseases, Imperial College Healthcare NHS Trust, London, London, UK; David Price Evans Infectious Diseases & Global Health Group, University of Liverpool Faculty of Medicine, Liverpool, UK; Department of Infectious Disease, Imperial College London, London, UK

## Abstract

**Background:**

Development of clinical decision support systems (CDSS) has been ongoing for over 60 years, more recently leveraging technologies such as artificial intelligence (AI) and machine learning (ML). Intelligent CDSS addressing different stages of the infection management process offer potential advantages in interpreting complex data and guiding clinical decision-making.

**Objectives:**

We outline the current applications of AI–driven CDSS across the continuum of bacterial infection management, from prevention and diagnosis to antibiotic prescribing and treatment individualization. We discuss the main limitations hindering their translation into clinical practice, as well as opportunities to improve their development to better meet clinical needs.

**Methods:**

References for this review were identified through searches of PubMed, Google Scholar, bioRxiv and arXiv up to March 2025 by use of a combination of ML, decision-making and bacterial infection keywords.

**Key findings:**

AI-CDSS studies increasingly leverage multimodal electronic health record (EHR) data, with most adopting lower-complexity models that perform well on structured data, particularly when supported by effective feature engineering. Despite efforts to develop accurate AI–driven systems, some of which achieve clinician-level accuracy in solving diagnostic and prescribing tasks, AI-CDSS have largely failed to integrate into clinical settings. Their adoption faces challenges related to the narrow scope of the defined medical task, failure to consider stakeholder workflow and lack of proper evaluation frameworks.

**Conclusion:**

There is a need to shift CDSS development towards a more adaptive and holistic approach that recognizes the continuous nature of the decision-making process in infection management. Comprehensive AI–powered platforms that can model infection dynamics could improve antibiotic stewardship and help tackle the global health emergency of antimicrobial resistance.

## Introduction

Artificial intelligence (AI) is projected to transform healthcare,^1,2^ with many potential applications in infectious disease (ID).^3^ AI methods have been developed across the ID continuum to improve outbreak detection, infection prevention and control and clinical management.^4^ By enhancing timeliness and accuracy, and by supporting personalized approaches to infection prevention and management strategies, AI tools offer unprecedented opportunities to address urgent challenges such as antimicrobial resistance (AMR).^5^

The prevention, diagnosis and treatment of bacterial infection is a complex, multi-step process that demands careful consideration of biological, economic and societal factors.^6,7^ Successful management of infection involves a sequence of interconnected steps with diagnostic and therapeutic decisions having both immediate and long-term potential consequences on individual patients and wider society. In this decision-making sequence, potential risk for errors can compound over time, affecting the patient’s overall outcome.^8^

Recent digitalization of patient health records in hospital settings has led to an increase in the volume and granularity of clinical data that is available for continuous monitoring and analysis over time.^9^ The heterogeneity of data used to represent and assess a patient’s health status introduces additional complexity, making it difficult for physicians to accurately derive useful clinical insights in a timely manner.^10^  ^,11^ There has been limited success in translating this wealth of high-dimensional patient data into disease insights and clinical decision guidance for the treatment of bacterial infection at the individual patient level. Research bottlenecks include data quality and complexity issues, lack of data standardization, privacy and ethical constraints and inability to deploy technology into real-world practice.^12^

Antibiotic stewardship programmes were established to support evidence-based management of bacterial infections and promote a coordinated approach to infection control through the development of guidelines tailored to local resistance patterns and resource availability.^11^ However, these rule-based interventions may fail to capture the nuances of individualized treatment and precision medicine. In addition, behavioural factors such as the influence of team hierarchy and the overreliance on clinical intuition often lead to deviations from guideline recommendations.^6^ Consequently, clinical decisions in bacterial infection management that have major implications on patient outcomes, particularly regarding antibiotic selection and timing, tend to be sub-optimal and disjointed, with the ‘right’ course of action often remaining unclear. In this context, there are significant opportunities for the development of data-intensive AI clinical decision support systems (AI-CDSS) to improve the accuracy, timeliness and individualization of antibiotic prescribing through the systematic integration of clinical knowledge and analysis of patient data.^11^

AI is a branch of computer science focused on creating systems capable of performing tasks that typically require human intelligence, such as learning, reasoning, problem-solving, decision-making, creativity and autonomy. The earliest use of AI in healthcare is in the form of expert systems (ESs), which condensed the knowledge of human experts into a series of if/then rules, enabling computers to mimic clinical reasoning.^8,9^ These rule-based systems formed the foundation of traditional CDSS, which aimed to standardize care by embedding clinical guidelines and logic into digital tools. Despite advantages in performance and explainability, traditional CDSS remain merely a reflection of the current state of knowledge delivered in a digital format, inherently not going beyond the capacity of clinicians to make informed decision.

The advent of machine learning (ML) marked a shift in AI–driven CDSS. ML models are designed to independently and continuously learn from data without being explicitly programmed to solve a particular task. Unlike traditional CDSS, ML–based CDSS do not rely on static rules, but instead use real-world data to learn underlying patterns, integrating varied sources of patient information to generate personalized insights.^10^ Such systems can use less structured, large-scale inputs to support clinicians with informed recommendations that evolve with new evidence, offering a more dynamic and scalable approach to clinical decision-making.^5^ While traditional CDSS systems based on static rules can technically be considered AI, we will refer to more recent, ML–based models as AI–powered CDSS for clarity.

**Figure 1.**
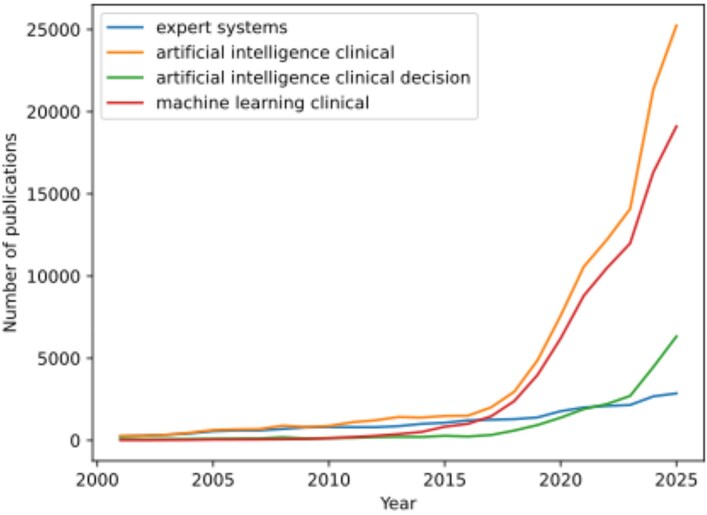
Growth in publications related to AI and clinical decision support over time. PubMed, 2000–25.

The rapid growth in publications applying AI and ML to clinical decision support, illustrated in Figure 1, underscores the accelerating interest and development in this field.

**Figure 2.**
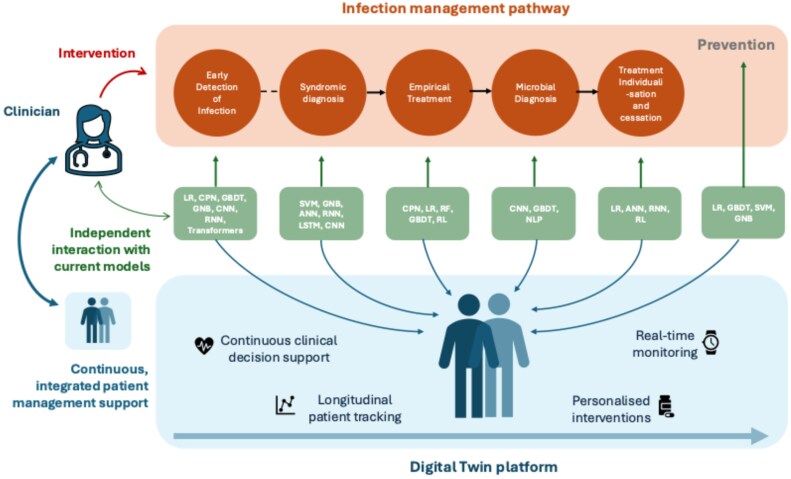
Schematic representation of the infection management process. The figure highlights the sequential stages of treatment and the role of CDSS. Traditional CDSS algorithms operate in isolation, addressing specific tasks such as empirical treatment, microbial diagnosis and treatment individualization. In contrast, a Digital Twin platform provides continuous, integrated support across all stages, dynamically updating based on patient data and clinical decisions. This real-time, evolving model of the patient enables a more holistic and adaptive approach to infection management.

This review examines how AI may transform clinical decision-making in the management of bacterial infection. We explore its potential to prevent infection and optimize antibiotic prescribing in secondary care, at each stage of the infection management pathway (Figure 2). We describe the architecture and performance of state-of-the-art AI models developed for clinical applications in bacterial infection. We consider the technical limitations and practical challenges to implementation of these AI tools in hospital settings. Finally, we argue that future progress depends on integrating AI tools across the full sequence of clinical decisions to enhance patient care, strengthen clinical judgement and improve infection outcomes.

## Methods

This narrative review aims to provide a broad overview of AI models that can support the management of bacterial infection.

### Search strategy

The literature search strategy aimed to identify articles describing the use of AI tools for managing bacterial infections. References were identified through searches of PubMed, Google Scholar, bioRxiv and arXiv for articles by use of a combination of ML keywords (‘deep learning’, ‘artificial intelligence’, ‘machine learning’, ‘neural networks’, ‘probabilistic networks’, ‘knowledge representation’, ‘digital twins’), decision-making keywords (‘medical decision’, ‘decision tool’, ‘support tool’, ‘clinical decision’, ‘CDSS’, ‘clinical management’, ‘decision making’) and bacterial infection management keywords (‘antibiotic’, ‘antibacterial’, ‘treatment’, ‘empirical treatment’, ‘treatment individualization’ ‘diagnosis’, ‘syndromic diagnosis’, ‘microbial diagnosis’, ‘sepsis’, ‘sepsis detection’, ‘prevention’, ‘infection prevention’). We included articles and relevant literature references cited in the selected publications up to March 2025.

### Study selection

Prospective and retrospective articles in English that reported original research on AI-CDSS for bacterial infection were included. We included development reports, implementation studies, clinical trials or qualitative studies in secondary care including intensive care units (ICUs). We excluded studies focusing on non-bacterial infections, paediatric studies and studies using non-clinical data.

### Definition of AI and ML methods

AI encompasses computational techniques that enable machines to perform tasks that typically require human intelligence, such as learning, reasoning and decision-making. Within AI, ML methods represent algorithms that can learn from data to identify patterns or predict outcomes without explicit programming. ML techniques can be classified into supervised learning, where algorithms learn the relationship between input features and known results using labelled data; unsupervised learning, in which methods analyse unlabelled data to uncover hidden patterns or structures without predefined outcomes; and reinforcement learning (RL) algorithms, which learn optimal actions through interaction with an environment and feedback in the form of rewards or penalties. Other AI methods such as natural language processing (NLP) and causal probability networks (CPNs) address specific types of data and reasoning tasks. We detail specific methods meeting this definition in Table 1.

**Table 1.** AI and ML techniques used in studies for this review

AI and ML technique	Description
Logistic Regression (LR)	A linear model commonly used for binary classification problems
Decision trees	A tree-based model that splits data into branches based on feature values to aid decision-making
Random Forest (RF)	An ensemble of decision trees that improves prediction accuracy and reduces overfitting
Gradient-boosted decision trees (GBDTs) (e.g. XGBoost)	An ensemble technique that builds trees sequentially, each correcting the errors of the previous ones
Support Vector Machine (SVM)	A model that identifies the optimal decision boundary separating the data into distinct classes
Gaussian Naive Bayes (GNB)	A probabilistic classifier based on Bayes’ theorem, assuming feature independence
Causal Probabilistic Networks (CPNs)	Probabilistic graphical models that represent and infer causal relationships among variables
Artificial neural networks (ANNs)	Computational models composed of interconnected nodes (‘neurons’) organized in layers, which learn complex patterns from data
Recurrent Neural networks (RNN)	Neural networks that capture temporal dependencies in sequential data by maintaining memory of previous inputs
Long Short-Term Memory (LSTM)	A type of RNN that can capture long-term dependencies in sequential or time-series data
Graph Neural Networks (GNNs)	A neural network designed to learn from graph-structured data (e.g. patient networks or molecular interactions)
Convolutional Neural Networks (CNNs)	Deep learning models designed to recognize spatial patterns, commonly used in image analysis
Reinforcement Learning (RL)	A learning framework in which an agent optimizes its actions through feedback from rewards and penalties
Transformers	Deep learning architectures using attention mechanisms to model complex dependencies, widely applied in text and sequence modelling
Natural Language Processing (NLP)	A field of AI focused on enabling machines to understand, interpret and generate human language

### Analysis of data in the selected articles

For each selected study, we extracted the variables used for model development, the algorithm architecture and metrics used for model evaluation. We split patient variables into microbiology (e.g. isolated organism and AST), pathology data (e.g. creatinine and white blood cell count), vitals (e.g. temperature and heart rate), demographics (e.g. age, sex and ethnicity), medical history (e.g. procedures, comorbidities and medications) and clinical interventions. ML architectures used for CDSS model development along with a brief description of their mechanism were summarized.

## Results

Table 2 summarizes literature identified as part of our search. In total, 37 papers published between 2006 and 2025 were identified. These were classified as focusing on syndromic diagnosis of infection, early detection of infection (mainly sepsis), empiric treatment selection, microbial diagnosis, treatment individualization and cessation, prevention of infection and digital twins (DTs).

**Table 2.** Summary of included AI-CDSS studies across the bacterial infection management pathway

Topic	Publication	Title	Study type	Year	Output	Population/settings	Data	ML algorithm	Evaluation
Syndromic diagnosis	Rawson *et al.*	Supervised machine learning for the prediction of infection on admission to hospital: a prospective observational cohort study	Prospective observational cohort study	2019	Bacterial infection diagnosis	Adult inpatients	Microbiology, pathology	SVM	Performance (AUROC)
Syndromic diagnosis	Rawson *et al.*	Supervised machine learning to support the diagnosis of bacterial infection in the context of COVID-19	Retrospective observational cohort study	2021	Secondary bacterial infection	Adult inpatients, hospitalized with positive COVID-19 PCR results	Demographic, pathology, microbiology	GNB, SVM, ANN	Performance (AUROC)
Syndromic diagnosis	Nigo *et al.*	Deep learning model for personalized prediction of positive MRSA culture using time-series electronic health records	Retrospective observational cohort study	2024	MRSA culture positivity	Adult patients, ED, IMU and ICU	EHR time-series data (demographics, microbiology, medical history)	Deep learning (RNN)	Performance (AUROC)
Syndromic Diagnosis	Ming *et al.*	Utilising routinely collected clinical data through time series deep learning to improve identification of bacterial bloodstream infections: a retrospective cohort study	Retrospective cohort study	2025	Prediction of blood culture results	Patients of all ages and care settings	Demographic, pathology	LSTM	Performance (AUROC, AUPRC)
Syndromic diagnosis	Karolcik *et al.*	Towards a machine-learning assisted non-invasive classification of dengue severity using wearable PPG data: a prospective clinical study	Prospective observational study	2024	Clinical risk stratification in dengue patients	Adult and paediatric patients with clinical diagnosis of dengue	PPG waveforms	CNN	Performance (precision and recall)
Early detection of infection	Shimabukuro et al.	Effect of a machine learning-based severe sepsis prediction algorithm on patient survival and hospital length of stay: a randomised clinical trial	Randomised clinical trial	2017	Patient survival, hospital LOS	Adult ICU patients	Demographic, vital signs, pathology	LR and multivariate feature engineering	Clinical impact (change in average hospital LOS and in-hospital mortality)
Early detection of infection	Valik *et al.*	Predicting sepsis onset using a machine learned causal probabilistic network algorithm based on electronic health records data	Retrospective observational cohort study	2023	Prediction of sepsis onset (48 h window)	Adult patients in ICU	Demographics, laboratory, vital signs, prescribing	CPNs, GBDT	Performance (AUROC, AUPRC)
Early detection of infection	Calvert *et al.*	A computational approach to early sepsis detection	Retrospective observational cohort study	2016	Prediction of sepsis onset (3 h in advance of the first 5 h SIRS episode)	Adult patients in ICU	Demographic, vital signs	LR and multivariate feature engineering	Performance (AUROC)
Early detection of infection	Brown *et al.*	Prospective evaluation of an automated method to identify patients with severe sepsis or septic shock in the emergency department	Prospective observational cohort study (with retrospective model derivation)	2016	Prediction of severe sepsis or septic shock within 1 h of ED arrival	Patients admitted to ED	Vital signs, pathology	GNB	Performance (AUROC, PPV, NPV)
Early detection of infection	Desautels *et al.*	Prediction of sepsis in the intensive care unit with minimal electronic health record data: a machine learning approach	Retrospective observational cohort study	2016	Detection of sepsis at onset and prediction of sepsis onset (1–4 h window)	ICU patients aged 15 years or more	Vital signs, Glasgow Coma Score and age	GBDT, LR	Performance (AUROC, AUPRC)
Early detection of infection	Mao *et al.*	Multicentre validation of a sepsis prediction algorithm using only vital sign data in the emergency department, general ward and ICU	Multicentre retrospective observational cohort study	2018	Prediction of sepsis, severe sepsis and septic shock	Adult patients, ED, general ward and ICU	Vital signs	GBDT	Performance (AUROC, AUPRC)
Early detection of infection	Ward *et al.*	Prediction of bacteraemia and of 30-day mortality among patients with suspected infection using a CPN model of systemic inflammation	Multicentre retrospective observational cohort study	2018	Prediction of probability of bacteraemia and 30-day mortality at the time of assessment	Adult patients with suspected infection, hospital setting (ward and ICU)	Vital signs, pathology data, demographics	CPN	Performance (AUROC)
Early detection of infection	Burdick *et al.*	Effect of a sepsis prediction algorithm on patient mortality, length of stay and readmission: a prospective multicentre clinical outcomes evaluation of real-world patient data from US hospitals	Prospective multicentre clinical outcome evaluation	2019	In-hospital mortality, LOS and 30-day readmission rate	Adult patients ED, general ward and ICU	Vital signs, demographics	XGBoost	Relative change in LOS, in-hospital mortality and 30-day readmission
Early detection of infection	Ward *et al.*	Automatic learning of mortality in a CPN model of the systemic inflammatory response syndrome	Retrospective observational model—development study	2017	Prediction of 30-day mortality and differentiation between sepsis and non-infectious SIRS	Adult patients with SIRS or suspected infection, hospital ward and ICU	Vital signs, pathology, demographics	CPN	Performance (AUROC)
Early detection of infection	Tang *et al.*	A time series driven model for early sepsis prediction based on transformer module	Retrospective observational cohort study	2024	Prediction of sepsis onset (12 h time window)	Adult patients in ICU	Demographics, pathology, vital signs (time-series)	CNN-Transformer and LSTM-Transformer	Performance (Accuracy, Precision, Recall, F1 score)
Early detection of infection	Xu *et al.*	Early prediction of sepsis using time series forecasting	Retrospective observational cohort study	2023	Prediction of sepsis onset (24 h time window)	ICU patients	Physiological time-series features, clinical text embedding (clinical BERT)	Self-supervised Transformer for Time-Series (STraTS), clinical BERT	performance (AUROC, masked MSE)
Early detection of infection	Zhou *et al.*	Interpretable machine learning model for early prediction of 28-day mortality in ICU patients with sepsis-induced coagulopathy: development and validation	Retrospective observational cohort study	2024	Prediction of 28-day mortality in ICU patients with sepsis-induced coagulopathy	ICU patients with sepsis-induced coagulopathy	Demographic, pathology, vital signs	XGBoost, SHAP	Performance (AUROC, AUPRC)
Early detection of infection	Li et al.	Real-time prediction of sepsis in critical trauma patients: machine learning-based modeling study	Retrospective observational cohort study	2023	Predict hourly risk of sepsis at lead-times of 4, 6, 8, 12 and 24 h before onset	Adult trauma patients admitted to the ICU	Demographic, pathology, vital signs	XGBoost, SHAP, feature engineering to derive patient features	Performance (AUROC)
Early detection of infection	Kim *et al.*	Development and validation of deep-learning-based sepsis and septic shock early prediction system (DeepSEPS) using real-world ICU data	Retrospective observational cohort study	2023	Prediction of sepsis and septic shock (24 h time window)	Adult patients in ICU	Demographic, pathology, vital signs	Deep learning (ANN, GRU)	Performance (AUROC)
Early detection of infection	Adams *et al.*	Prospective, multi-site study of patient outcomes after implementation of the TREWS machine-learning-based early warning system for sepsis	Prospective observational cohort	2022	Association between clinician response to TREWS alerts and patient outcomes (mortality, organ failure, LOS)	Adult inpatients (ED, wards, ICU)	Real-time EHR data (vitals, labs, demographics, antibiotic orders)	GBDT	Adjusted mortality reduction
Empirical treatment	Paul *et al.*	Improving empirical antibiotic treatment using TREAT, a computerized decision support system: cluster randomized trial	Cluster randomized controlled trial	2006	Appropriate empirical antibiotic treatment, antibiotic costs, broad-spectrum usage, length of stay, mortality	Patients suspected of bacterial infection in hospital wards	Microbiology, antibiotic prescriptions	CPN	Antibiotic treatment appropriateness
Empirical treatment	Stracy *et al.*	Minimizing treatment-induced emergence of antibiotic resistance in bacterial infections	Retrospective observational cohort study	2022	Prediction of treatment-induced antibiotic resistance risk	Adult patients with recurrent bacterial infections	Microbiology, antibiotic exposure history and genomic data	LR	Performance (AUROC)
Empirical treatment	Corbin *et al.*	Personalized antibiograms for machine learning driven antibiotic selection	Retrospective observational cohort study	2022	Prediction of antibiotic susceptibility patterns	Adult patients in ED	Demographic, pathology, vital signs, microbiology, patient comorbidities	LR, RF, XGBoost	Performance (AUROC, antibiotic coverage rates relative to clinician choice)
Empirical treatment	Komorowski *et al.*	The artificial intelligence clinician learns optimal treatment strategies for sepsis in intensive care	Retrospective observational cohort study	2018	Learned policy for IV fluids + vasopressors optimizing 90-day survival in sepsis	Adult ICU patients meeting Sepsis-3 criteria from	Demographic, pathology, vital signs	RL using Markov decision process (MDP)	Relative AI policy value on patient mortality
Microbial diagnosis	Eickelberg *et al.*	Development and validation of MicrobEx: an open-source package for microbiology culture concept extraction	Retrospective observational cohort study	2022	Prediction of microbiology culture result	ICU patients	Free text microbiology reports	NLP	Performance (F1 score)
Microbial diagnosis	Smith *et al.*	Automated interpretation of blood culture gram stains by use of a deep convolutional neural network	Retrospective observational cohort study	2018	Automatic classification of Gram stain images	Hospital inpatients	Gram-stained slides	CNN	Performance (AUROC)
Microbial diagnosis	Nguyen *et al.*	Developing an in silico minimum inhibitory concentration panel test for Klebsiella pneumoniae	Retrospective observational cohort study	2018	Prediction of antibiotic MICs	Hospital inpatients	Whole genome sequences	XGBoost	Performance (accuracy)
Microbial diagnosis	Miller *et al.*	Deciphering microbial gene function using natural language processing	Retrospective observational cohort study	2022	Embedding-based gene function classification	Adults	Whole genome sequences	NLP	Performance (accuracy, precision, recall, F1 score)
Treatment individualization and cessation	Bolton *et al.*	Personalising intravenous to oral antibiotic switch decision making through fair interpretable machine learning	Retrospective observational cohort study	2024	Prediction of suitability/timing for switch from IV to oral antibiotics	Adult patients in ICU	Pathology, vital signs, antibiotic treatment	ANN, SHAP	Performance (AUROC, F1 score)
Treatment individualization and cessation	Bolton *et al.*	Machine learning and synthetic outcome estimation for individualised antimicrobial cessation	Retrospective observational cohort study	2022	LOS and mortality	Adult ICU patients receiving IV antibiotics	Pathology, vital signs, antibiotic treatment	RNN autoencoder (bidirectional LSTM)	RMSE, mean LOS reduction, mortality reduction
Treatment individualization and cessation	Beaudoin *et al.*	Evaluation of a machine learning capability for a clinical decision support system to enhance antimicrobial stewardship programs	Prospective evaluation study	2016	Identification of inappropriate antimicrobial prescriptions	Adult inpatients	Demographic, pathology, vital signs, microbiology, patient comorbidities, locations	Not specified	Inappropriate antibiotic prescription rate
Treatment individualization and cessation	Ellligsen *et al.*	Improving decision making in empiric antibiotic selection (IDEAS) for gram-negative bacteremia: a prospective clinical implementation study	Prospective clinical implementation study	2021	Facilitation of early de-escalation of antimicrobial therapy	Adult ICU patients	Demographic, microbiology, medical history	LR and multivariate feature engineering	Number of switching interventions
Treatment individualization and cessation	Boominathan *et al.*	Treatment policy learning in multiobjective settings with fully observed outcomes	Retrospective observational cohort study	2022	Selection of antibiotic treatment policies for UTIs	Adult women with UTIs	Demographics, microbiology, antibiotic prescriptions, pathology, medical history	RL	Inappropriate antibiotic therapy rate
Prevention	Jakobsen *et al.*	Clinically explainable machine learning models for early identification of patients at risk of hospital-acquired urinary tract infection	Retrospective observational cohort study	2024	Prediction of HA-UTI risk	Adult inpatients	Demographics, vital signs, medical history, pathology	LR, BN, DTC, ANN, RF, AdaBoost classifier (AD), GBDT, SHAP	Performance (AUROC)
Prevention	Hernandez *et al.*	Supervised learning for infection risk inference using pathology data	Retrospective observational cohort study	2017	Prediction of infection risk	Adult inpatients	Microbiology, pathology	GNB, SVM, DTC, RF	Performance (AUROC)
Prevention	Myall *et al.*	Prediction of hospital-onset COVID-19 infections using dynamic networks of patient contact: an international retrospective cohort study	Retrospective observational cohort study	2022	Prediction of individual risk of hospital-onset COVID-19 infection	Hospital inpatients	Demographics, dynamic in-hospital bed allocation and movement, virology results	XGBoost	Performance (AUROC)
DTs	Lal *et al.*	Development and verification of a digital twin patient model to predict specific treatment response during the first 24 h of sepsis	Retrospective observational cohort study	2020	Treatment response during first 24 h of sepsis	Adult ICU patients	Vital signs, pathology, treatment interventions	Hybrid DT combining agent-based simulation, discrete-event simulation, and BN	Performance (agreement between observed and expected responses)

For each study, the table summarizes the topic, publication details, study type, year, model output, study population and clinical setting, input data, underlying ML architecture and reported evaluation metrics.

AUROC, area under the receiver operating characteristic curve; AUPRC, area under the precision–recall curve; BN, Bayesian network; CPN, causal probabilistic network; ANN, artificial neural network; SVMs, support vector machines; RNN, recurrent neural network; LOS, length of stay; LSTM, long short-term memory; GNB, Gaussian Naive Bayes; PPG, photoplethysmogram; CNN, convolutional neural network; LR, logistic regression; GBDT, gradient boosting decision tree; XGBoost, extreme gradient boosting; GRU, gated recurrent unit; RF, random forest; NLP, natural language processing; MDP, Markov decision process; RCT, randomized controlled trial; SHAP, SHapley Additive exPlanations; SIRS, systemic inflammatory response syndrome; SIC, sepsis-induced coagulopathy; DT, digital twin.

### Syndromic diagnosis

Diagnosing medical conditions is a fundamental challenge in clinical practice.^13^ In ID, diagnosis involves the identification of the causative pathogen and the precise site of infection. General syndromic diagnosis obtained through physical examination and analysis of clinical symptoms is often conducted in the early stages of infection management. Following initial assessment, the precise identification of the causative pathogen is often achieved through microbiological culture, nucleic acid amplification tests such as PCR or serological testing.

The primary challenge in diagnosing bacterial infection lies in integrating the information derived from the initial clinical evaluation and subsequent microbiological results as these are typically carried out at different time points and require distinct, often non-overlapping, areas of clinical expertise. Relevant data are often stored in separate systems, hindering data linkage and limiting clinicians’ ability to build a comprehensive, real-time overview of the patient’s infectious status.^14^

AI-CDSS are being explored to bridge the gap between syndromic diagnosis and phenotypic confirmation by supporting more accurate diagnosis during the early stages of infection management, thereby guiding more targeted therapy while definitive diagnostic results are being processed.^3,15^

A common approach for early prediction of positive microbiology cultures involves the use of static classifiers such as logistic regression or support vector machines (SVMs), which leverage patient features derived from electronic health records (EHRs) to predict outcomes. This method has been applied to applications including the identification of secondary bacterial infections in COVID-19–positive patients^16^ and the detection community-acquired infections at the time of admission.^17^ Reported performance varied, with an area under receiver operating characteristic (AUROC) of 0.91 for predicting positive microbiological samples within 48 h of admission^16^ and 0.84 for infection diagnosis within 72 h.^17^

While static models provide a baseline for early infection prediction, they are most effective close to the time of diagnosis, functioning more as detection tools than true prediction models. Their inability to appropriately weight timings of clinical events limits their capacity to capture the temporal dynamics of patient deterioration, often limiting predictive accuracy. Dynamic methods, such as long short-term memory (LSTM) networks, a type of recurrent neural network (RNN), address the limitations of static models by processing sequential data and retaining information across time steps. This enables them to learn temporal dependencies and recognize patterns in the progression of clinical variables such as the timing and order of symptoms or laboratory results, improving performance and forecasting range.

One study predicted positive MRSA cultures up to a fortnight in advance of sample collection using an RNN, achieving an AUROC of 0.91.^18^ Another study employed a bidirectional LSTM to predict positive blood culture outcomes, also up to 2 weeks in advance and achieving an AUROC of 0.99.^19^ In a further example, the potential of incorporating time series data on model performance was demonstrated with the authors improving a static regression model (AUROC 0.74) prediction using LSTM (AUROC 0.97) to diagnose blood stream infections with data up to 14 days before blood sample collection.^20^

Continuous physiological monitoring through wearable technology to extract patient features over time can be used for infection diagnosis prior to symptom onset.^21^ A range of examples for this approach were piloted during the COVID-19 pandemic and include using wearable technologies to monitor heart rate, respiratory rate, cough frequency and walk cadence.^22^ Another approach used convolutional neural networks (CNNs), a class of deep learning models effective for image analysis, to predict dengue fever with photoplethysmogram (PPG) waveforms.^23^ For bacterial infection, wearable sensors and continuous monitoring of vital signs have been investigated to detect early signs of sepsis and other bacterial illnesses.^24^ ML models applied to these longitudinal physiological data can help identify patterns indicative of bacterial infection before clinical symptoms become apparent and without the need for invasive procedure.^25^

### Early infection detection

A major area of focus for AI-CDSS in bacterial infection to date has been the early detection and prediction of sepsis, a condition where delayed diagnosis can have severe consequences. Sepsis is a dysregulated host response to infection associated with life-threatening organ failure.^26^ An estimated 11 million sepsis-related deaths were recorded worldwide in 2017, accounting for approximately 20% of all global deaths.^27^ Given the association between treatment delays and mortality, sepsis guidelines emphasize the importance of initiating appropriate antimicrobial therapy within 1 h of onset of septic shock.^28^

Traditionally, sepsis detection relied on the manual screening and evaluation of patient parameters against established scoring systems such as SIRS, SOFA and NEWS.^28^ While manual screening continues in many settings globally, automated computation of sepsis scores through digital sepsis alerts (DSAs) integrated into EHR systems has become widespread in most secondary care settings of high-income countries.^29^ DSAs monitor patient data and notify healthcare professionals when relevant clinical parameters deviate from their normal ranges, signalling patient deterioration.

While some studies report improvements in patient outcome (e.g. mortality, length of stay and ICU transfer) and process measures (e.g. time to antibiotic administration and lactate measurement), evidence for overall clinical benefit of rule-based DSAs remains mixed.^30–33^ In addition, these warning scores are primarily designed to detect rather than predict sepsis, with clinical suspicion often arising before alerts trigger.^34^ DSAs provide a useful foundation for sepsis screening, though they remain relatively blunt and non-specific.^35^

Given the high mortality rate associated with sepsis and the potential of earlier interventions in reducing mortality, it is perhaps unsurprising that many AI-CDSS development efforts focused on sepsis prediction and patient stratification.^30,36–43^ There is now evidence that ML–based systems may leverage patient data more effectively, improving the precision, accuracy and timeliness of sepsis prognosis compared to rule-based methods.^44^

A range of AI methodologies have been explored for sepsis prediction. Gradient tree boosting (GTB) models have been extensively studied due to their ability to capture complex relationships between clinical variables. One notable example is *InSight*, a GTB algorithm that uses feature engineering to analyse high-order correlations of patient vitals and capture the temporal dynamics of multi-organ systems.^39^ Transfer learning on external, open-access datasets such as the Medical Information Mart for Intensive Care (MIMIC) dataset^45^ was used to enhance model performance and overcome local healthcare data limitations.^40^

InSight outperformed traditional scoring systems at the time of sepsis onset in the ICU (AUROC 0.88).^40^ However, alerts triggered at onset provide limited clinical benefit, as patients already meet criteria recognizable by clinicians. InSight’s key utility lies in its predictive capability, achieving an AUROC of 0.85 for predicting severe sepsis 4 h before onset, thereby enabling earlier clinical intervention. In a single-centre prospective interventional trial, its use was associated with reduced ICU length of stay and in-hospital mortality.^35^

An alternative algorithm, SepsisFinder, was able to predict sepsis onset within a 48 h period with an AUROC of 0.95 using sparse EHR data.^36^ SepsisFinder uses causal probabilistic networks (CPNs), a type of AI algorithm extensively studied for infection diagnosis due to simplicity, interpretability and ability to handle missing data natively.^46,47^ The similarity of CPNs to clinical reasoning makes these models highly interpretable, an important factor in developing trust in AI–powered systems.^48^ SepsisFinder was developed by refining parts of the established TREAT network,^49^ highlighting the adaptability of AI algorithms to accelerate progress towards next-generation solutions in ID.^36,41^  ^,46^

Time-series AI models that continuously integrate longitudinal patient data represent a promising approach for real-time sepsis prediction. Transformer-based architectures such as those developed by Tang *et al.*^50^ model long-range dependencies in vital signs, achieving accuracies above 95% up to 12 h before diagnosis. Similarly, Xu *et al.*^51^ combine self-supervised learning and multimodal inputs including time-series data and clinical text embeddings to predict sepsis up to 24 h in advance with an AUROC of 0.89.

While current AI models show strong performance for sepsis prediction using clinical data derived from ubiquitous monitoring devices, it is difficult to estimate pooled performance due to the wide heterogeneity in sepsis definition, data preprocessing and the chosen prediction time window prior to sepsis onset. The patient inclusion criteria for models processing temporal data often require a minimum of several hours of variables before model predictions can be made, which limits applications in a real-life hospital setting.^40^ The trade-off between accuracy and timeliness emerged as a limitation in time-series processing for sepsis detection.^39^

Additionally, a wide range of sepsis models were developed based on ICU data.^44,50–54^ While the wealth of high-granularity data continuously recorded over time provides an opportunity for the development of data-intensive AI models, the ICU may not be the optimal target for AI-based interventions. Some studies reported that sepsis prediction tools provide little improvement in patient outcomes in the ICU compared to their application in hospital wards and emergency departments.^44^ This discrepancy is likely related to a ceiling effect, given that the ICU environment already involves intensive monitoring and frequent clinical interventions. CDSS impact may be greater in hospital-wide settings where inappropriate prescribing is more common. Nonetheless, the ICU remains a valuable environment for model development and prospective validation before broader deployment.

From a clinical standpoint, the most useful models would continuously update and provide real-time predictions of sepsis risk enabling timely clinical interventions. Clinical outcome studies provide some evidence that the deployment of AI–powered warning systems are associated with a reduction of in-hospital mortality and average hospital length of stay.^35,43,55^ Despite the inherent limitations including surveillance bias, changes in treatment standards over time and lack of randomization, prospective observational studies provide important initial insights into the potential of AI tools in real-world applications.

### Empirical treatment

Antibiotic prescribing, whether appropriate or inappropriate, is a major driver of AMR, a global health emergency projected to be responsible for approximately 10 million deaths a year by 2050.^56^

During the early stages of infection management, treatment decisions are often influenced by the lack of available information with regard to the patient’s health status, time constraints, cognitive limitations and fatigue.^11^ Optimal treatment involves striking the balance between positive patient outcomes, minimizing side effects and reducing the risk of resistance emergence. Clinical intervention to reduce suffering and hamper disease progression is imperative, and concerns over the management of individual patients often promote conservative therapy using broad-spectrum agents prior to identification of the causative organism and its susceptibility profile.^57^

This precautionary attitude to minimize treatment failure drives over-prescription of unnecessarily broad-spectrum antibiotic therapy.^58^ The overuse of broad-spectrum agents is associated with an increased risk of selecting AMR,^59^ serious adverse events such as nephrotoxicity, *Clostridioides difficile* infection, disruption of normal gut microbiota^60^ and potentially inferior clinical outcomes.^61^

Prescribing is carried out in large part by non-experts in infection management who may have a limited understanding of the mechanisms that drive the development of resistance.^6^ To support empiric prescribing decisions, guidelines are designed to be broadly applicable to a patient population but frequently fail to consider individual patient features. Behavioural aspects such as influence of peers, the existence of unspoken rules related to antibiotic management (‘prescribing etiquette’), fatigue and clinical intuition-driven decision-making limit the effectiveness of empiric treatment guidelines in promoting optimal infection management.^11,57^

Computerized decision support for empirical antibiotic selection stems from ESs such as MYCIN.^62^ MYCIN was developed based on rules captured from interviews with medical experts and showed good performance in a small prospective study for therapy selection in patients with positive blood cultures.^62^ Despite its theoretical potential, systems such as MYCIN have failed in the implementation stage as they are inherently rigid and not adaptable to the dynamic nature of local resistance patterns and antibiotic availability.^63^

An example of an early AI–driven approach for antibiotic selection is the TREAT algorithm^49^ that uses CPNs to predict clinical events in patients with systemic infection.^47^ CPNs are somewhat reminiscent of ESs as they combine expert knowledge with data-derived clinical insights. In a clustered randomized trial, TREAT prescribed appropriate empirical treatment more frequently than clinicians while also reducing the antibiotic spectrum and treatment associated costs.^49^ However, physician compliance remained a major barrier to implementation as algorithm recommendations were not always applied.

Currently, recognition that matching the pathogen’s susceptibility profile is not sufficient in ensuring treatment efficacy is becoming a foundational assumption in CDSS development. Treatment recommendations must also weigh the trade-offs between optimizing patient outcomes, preventing resistance or recurrence at the individual level and preserving the efficacy of antibiotics at a societal level by considering how frequently a given agent can be prescribed without accelerating resistance. AMR often occurs through reinfection with a resistant strain from the patient’s own microbiota rather than through *de novo* resistance evolution, highlighting the predictable nature of resistance at the individual-patient level.^59^ AI-CDSS therefore focus on predicting the risk of treatment-induced resistance to ensure treatment safety and efficacy. Logistic regression models show high performance in predicting resistance based on patient EHR data.^59,64^ ML algorithms identified an alternative agent for most susceptibility-matched treatments prescribed by clinicians, reducing the risk of resistance by 70%.^59^

A similar study used ML techniques [logistic regression, random forests (RFs) or gradient boosted trees] to generate personalized antibiograms from EHR data.^64^ Personalized antibiograms were subsequently used to reallocate antibiotics across a set of patients in a way that maintained or improved the coverage rates achieved by clinicians while using narrower spectrum antibiotics.^64^ While individual model performance was relatively poor (AUROC 0.61–0.73), algorithm treatment recommendations matched or exceeded clinician performance, suggesting that proxy measures such as AUROC alone are not always direct indicators of clinical utility.^65^

This discrepancy could also reflect the inherent bias of medical data and the potential shortcomings in our current ability to select the most appropriate treatments for patients.

Simple ML models could therefore provide solutions for personalized care while also uncovering underlying patterns in AMR development. A major limitation of ML algorithms is in their reliance on patient metrics, which may not be readily available such as prior antibiotic use.^59^ The importance of medical history in determining treatment success highlights the need for continuous patient tracking and logging of clinical decisions over time and across levels of care. This is a salient example of how individual decisions in the infection management pathway can have far-reaching consequences on the individual patient and the society level.

The use of AI for clinical interventions beyond antibiotic management is shown in the study by Komorowski *et al.*^66^ in which a RL model supported sequential decision-making problems in sepsis. The performance of the RL model reflects the potential of AI–driven treatment recommendations and provides an example of the need to shift CDSS towards more holistic approaches to patient management. The RL model inferred optimal treatment from real-life sub-optimal training examples of vasopressor and intravenous fluid administration, learning a treatment policy that matches that of human clinicians that favoured patient survival at Day 90.

### Microbial diagnosis

Culture-based diagnostics remain the gold standard for identifying bacterial pathogens and guiding antibiotic treatment through phenotypic antibiotic susceptibility testing (AST). Culture-based diagnostic workflows are inherently time-consuming, with laboratory results only available 24–48 h after sampling due to the need for organism incubation and growth.^67^ This turnaround time (TAT) can lead to clinical uncertainty during the critical early phases of treatment, leading to empirical treatment that may be sub-optimal.

One promising approach to addressing delays in the TAT of microbiology culture reporting involves the use of AI applications focused on the interpretation of microbiology culture, image and report data, which are often complex and heterogeneous in nature, and the analysis of which is typically labour intensive and requires specialized expertise.^68^ Recent advances in NLP, a subfield of AI focused on interpreting human language, have enabled the development of models that can extract information from unstructured clinical notes and integrate various data types to maximize the use of available patient information. In clinical metagenomics, NLP models have been used to automatically interpret microbiology reports, determining whether a culture is positive and returning a list of SNOMED-CT–mapped bacterial identification codes.^69^ Another example of the application of AI for the analysis of data-rich microbiology information is the use of CNNs, which automatically learn and extract hierarchical image features to recognize visual patterns. These models have achieved 93% accuracy in classifying whole-slide images of blood culture Gram stains.^70^

Molecular and genomic diagnostics have emerged as a powerful alternative to identify the causative organism without any need for culture, by sequencing DNA or RNA directly from clinical samples and allowing for faster and more comprehensive identification of pathogens. Additionally, metagenomics can detect antibiotic resistance determinants, identify previously undetected pathogens and provide insights in cases where traditional diagnostic workflows fail.

While rapid molecular diagnostic techniques are becoming increasingly available, reducing time to diagnosis and increasing precision compared to traditional culture-based methods, they often fail to provide a full phenotypic picture required for personalized antibiotic therapy.^71,72^ In addition, while they can identify genetic markers of pathogens or resistance, the resulting data are often complex and difficult to interpret in a clinical context where we still rely on AST to make targeted treatment decisions.^73^

Conventional culture-based AST requires 18–24 h to determine bacterial susceptibility, delaying treatment. AI and deep learning offer faster alternatives with one study using a CNN to analyse single-cell morphological phenotypes in *Escherichia coli*, achieving 80% single-cell accuracy in classifying susceptibility to four antibiotics (ciprofloxacin, gentamicin, rifampicin and co-amoxiclav) in just 30 min.^73^

WGS data of pathogens represent another complex data type increasingly integrated into clinical workflows. ML applied to WGS data in clinical microbiology has been used to explore the relationship between DNA sequence and AMR phenotypes. In one study, ML techniques were applied to infer resistance phenotypes for 16 antibiotics from WGS data of *Klebsiella pneumoniae* clinical isolates.^74^ Building on this, an extreme gradient boosting (XGBoost)-based model was developed to predict the MIC for 20 antibiotics based on *K. pneumoniae* WGS data.^75^ Notably, this approach required no *a priori* knowledge of resistance genes and delivered results in a format that is interpretable and practical for routine clinical care, unlike traditional gene-based resistance detection that often fails to directly inform antibiotic treatment decisions.^76^ Although WGS is not yet routinely available and typically follows conventional AST in diagnostic timelines, such models provide a valuable proof of concept for the potential of AI to analyse highly complex, high-dimensional genomic data and to ultimately inform or complement antibiotic treatment decisions as sequencing technologies become faster, cheaper and more integrated into real-time infection management workflows.

Given the similarity between genome sequences and natural language, advancements in transformer-based methods for NLPs could also accelerate genomic analysis to provide valuable insights into pathogen susceptibility profiles, thereby encouraging antibiotic stewardship and more targeted treatment strategies.^77,78^ For example, one study used an NLP model to predict gene functions based on 360 million microbial genes, uncovering novel microbial defence systems associated with resistance.^79^ There is therefore potential for integrating ML to enhance diagnostic accuracy and automate data interpretation.^80^ AI–driven tools have the potential to bridge the gap between molecular diagnostics and clinical decision-making, enabling faster and more precise actionable diagnosis of bacterial infections.

### Treatment individualization and cessation

Treatment is often individualized or discontinued as patients progress through the treatment sequence and additional information becomes available. Treatment individualization is often an important part of antibiotic stewardship, ensuring that prescriptions are both effective and judicious. This approach minimizes unnecessary exposure and side effects while addressing the patient’s specific clinical needs and preserving the effectiveness of existing antibiotic therapies for future generations.^8^

There is a relative paucity of research into AI technology to support the individualization or cessation of antibiotic treatment. Clinical practice in this area is often heterogeneous and non-standardized, with real-world research demonstrating variable results.^81–84^ In one AI–focused study, researchers employed gradient-boosted models to predict different aspects of antibiotic stewardship: treatment discontinuation, intravenous to oral switching (IVOS) and early and late transition to targeted, narrow-spectrum therapy.^85^ Models achieved an AUROC ranging from 0.72 to 0.81, with 41% more patients being deemed suitable for cessation compared to conventional fixed duration of treatment approaches.

ML–based models to predict IVOS were developed in another study.^86^ Researchers took the temporal nature of the patient’s infection into account, identified a small set of informative features and ensured their ML models were fair and interpretable, achieving an AUROC of 0.80 for IVOS.^86^ One study focused on understanding when to stop antibiotic treatment by introducing a novel AI strategy to estimate the potential impact of stopping or continuing treatment on both mortality and length of stay to address prescriber concerns that early treatment discontinuation may lead to poor patient outcomes.^87^ This approach demonstrated that some patients could have stopped treatment earlier without adverse effects.

An alternative approach to refining initial treatment decisions is to use a knowledge base and supervised learning approach to extract rules and ensure appropriate prescriptions of antibiotics, such as piperacillin–tazobactam.^88^ Other studies have touched on targeted antibiotic therapy without it being the primary research focus. For example, one study showed that a logistic regression model for predicting appropriate empiric therapy can facilitate early de-escalation by switching to narrow spectrum antibiotics.^89^

Antibiotic stewardship decisions are notoriously complex. Balancing the care of an individual patient with the public health priority of AMR is non-trivial and has not been solved in the context of AI-CDSS.^90,91^ The research presented above has limitations in that it often fails to consider behavioural factors with regard to prescribing, and models can be biased towards heterogenous clinical practice. Data-driven AI approaches can tackle treatment individualization and de-escalation, but the degree has yet to be fully established.

### Prevention

Preventing the spread of infection in healthcare settings is paramount in reducing the burden of hospital acquired infections (HAIs) and slowing the evolution of treatment-resistant pathogens.^92,93^ While basic measures such as handwashing, personal protective equipment and regular environmental cleaning are effective in preventing some transmission,^94^  ^,95^ a lack of detailed information on patient locations or diagnoses often leads to infections being detected too late for prevention interventions to be meaningful.^96^ By employing AI modelling techniques that integrate patient variables and inter-patient contact, the risk of infection propagation can be predicted and thereby prevented.

Targeted approaches to preventing HAIs focus on developing risk prediction models using patient biomarkers, similar to early diagnostic methods,^97,98^ but specifically addressing infections acquired during hospital admission. Urinary tract infections (UTIs), one of the most common HAIs, account for up to 30% of nosocomial infections.^99^ To predict hospital-acquired UTIs, one study trained a neural network for early identification, achieving an AUROC of 0.76 while also enhancing explainability by using SHapley Additive exPlanations (SHAP) values, to determine the most predictive variable.^97^

To forecast outbreaks of viral infections and better understand the transmission of HAIs, one study incorporated patient location data to build network graphs, enriching each person’s features.^100^ This approach significantly improved the prediction of COVID-19 cases in hospitals.^100^ Network graphs represent the relationships and interactions between patients by visualizing connections and movement patterns. Increased detail in patient movements can be achieved through real-time location systems, such as wearable radio frequency identification (RFID) tags, enabling the development of ML models that can pinpoint transmission events and facilitate targeted interventions.

The key difference between AI-CDSS focusing on prevention and those designed to optimize antibiotic prescribing is the level of systems-level details that are currently incorporated into models. These can include patient locations, which have significantly improved model performance and supported prediction of transmission between patients.^100^ The concept of using patient networks to understand transmission dynamics can also be applied to bacterial infections, by considering the interactions between patients who shared a bathroom or operating theatre.^101^ Additionally, understanding transmission within a patient network is crucial for identifying the spread of AMR.^102^ Preventing outbreaks of this nature is essential for slowing the development of resistant pathogens and reducing the selection pressure in healthcare settings and should therefore not be considered in isolation of other AI-CDSS being developed to optimize antibiotic prescribing.

## Discussion

### Key findings

Across the selected AI-CDSS studies, summarized in Table 2, the data modalities and scope have expanded beyond the vitals-only approach used in traditional scoring systems, to encompass multimodal EHR representations, combining demographics, pathology data, treatment and medication context and microbiology results. A smaller subset of studies further incorporated higher-dimensional inputs, such as WGS and free-text clinical documentation, reflecting a progressive trend towards richer patient representations.

The methodological landscape of AI-CDSS spans a wide range of model architectures, from interpretable probabilistic approaches [e.g. CPNs and Bayesian networks (BNs)], tree-based ensembles, gradient-boosting methods, deep learning models and hybrid frameworks integrating RL or DT simulations. In practice, lower-complexity models such as logistic regression (LR) and decision trees were most prevalent and generally achieved strong predictive performance. This choice of model reflects the fact that tabular EHR data, typically of moderate dimensionality, do not necessarily benefit from deeper architectures for analysis. Instead, methodological advances focused on feature engineering techniques to derive more informative predictors from routine clinical variables to capture latent interactions and feature dependencies and enhance model performance. In contrast, more complex deep learning architectures were typically reserved for higher-dimensional data modalities such as imaging, free-text clinical notes, genomic sequences or continuous physiological time series.

Validation maturity varied across studies. The majority remained retrospective cohort studies, often single-centre, with internal or limited external validation. A smaller subset demonstrates prospective, real-world deployment (e.g. TREWS) to study the clinical utility and impact of patient outcomes such as mortality and length of stay. Interpretability and algorithmic fairness increasingly emerged as priorities in model development, with several studies employing explainable-AI methodologies (e.g. SHAP) to support clinician trust and equitable performance.

### Current AI-CDSS limitations

AI-CDSS for infection management hold significant potential for improving patient care while alleviating the financial and workload burdens of healthcare systems. Their adoption remains limited compared to fields such as radiology and oncology, where decision support tools are more routinely integrated into clinical practice and several AI–based medical devices have received regulatory approval.^103,104^ The implementation of AI tools in these fields likely relates to the availability of standardized, high-quality imaging data (e.g. MRI, CT and histology), clearly defined outcomes and strong evidence of clinical benefit. In contrast, decision-making in ID involves heterogeneous, sparse and evolving clinical data and less clearly defined outcomes, which may partly explain the slower uptake of AI-CDSS. Successful translation of AI-CDSS into practice therefore requires careful consideration of several key challenges, including the technical limitations of AI models, practical barriers to real-world deployment and behavioural factors among stakeholders that may hinder adoption.^105^

### Data quality, bias and representativeness

As with most AI applications, model performance is highly dependent on the quantity and quality of available data. Currently, the development of AI–powered technologies in infection management is unevenly distributed across infection types, clinical decision time points and levels of care. ICUs benefit from high-volume, granular datasets and open-source resources such as MIMIC.^45^ In contrast, a paucity of models tailored to primary care settings and underrepresented populations including the elderly, pregnant women and infants are available reflecting the gaps in the data used for model development.

Disparities in health data availability between high- and low-resource settings present a significant barrier to the equitable use of data-driven digital technologies and prevent them from being sustainably delivered at scale.^12^ Without targeted efforts to address these imbalances, data poverty could pose a real danger in deepening existing healthcare inequalities by contributing to a growing digital health divide.^106^

It remains unclear whether EHR data alone are sufficient for developing robust models in infection disease management. There is a growing need for consensus on the fundamental patient variables that should be included during data collection, which may extend beyond routinely collected EHR data. These could include antibiotic exposure history, comorbidities and genomic information, all of which may be essential in enabling accurate data-driven decision-making.^12^ Overlooking such patient variables that are likely responsible for inter-individual variability in treatment outcomes can introduce unintended biases and lead to accidental fitting of confounders within models.^105,107^

Beyond data quantity and quality, the lack of standardization in EHR data structures and clinical coding practices across institutions limit interoperability, posing challenges for multicentre model training and validation. Socioeconomic disparities further contribute to bias, as most AI–based interventions are developed and validated using datasets derived from high-income countries, limiting generalizability and effectiveness in low-resource settings.^105,107,108^

### Model generalizability and validation

An important shortcoming inherent to AI algorithms is their limited applicability outside the training domain. Many AI-CDSS are developed using single-centre data that constrain their generalizability to different settings due to technical differences between sites (equipment, clinical coding systems and EHR infrastructure) as well as variation in patient demographics, clinical workflows and local epidemiology.^105^ Site-specific training is required to adapt AI models to new populations. Even within the same clinical setting, AI models trained on historical data are susceptible to performance degradation over time when exposed to evolving clinical environments. Data shifts caused by updates in medical devices, evolving clinical guidelines and changes in administrative practices can significantly affect model robustness and generalizability. To mitigate this decline in performance, a proposed strategy has been the periodic retraining or recalibration of these models on an annual basis to maintain reliability and clinical safety.^105,109^

Beyond generalizability concerns, evaluation approaches for AI-CDSS often provide an incomplete picture of clinical utility. Current AI-CDSS are often optimized for conventional metrics such as specificity, sensitivity and AUROC.^110^ While balancing sensitivity and specificity is crucial in healthcare, these metrics are proxy measures and do not directly reflect real-world clinical utility. This can be particularly misleading in the presence of class imbalance, an inherent characteristic of medical datasets.^108^ In such cases, the low prevalence of severe cases, which are typically the primary targets of prediction tools, may result in an inflated sense of performance when predominantly predicting non-severe cases. Alternative metrics such as the area under the precision–recall curve (AUPRC), F1 score and positive predictive value (PPV) offer more informative insights, particularly when evaluating a model’s ability to detect rare but clinically significant cases.^110^ In addition, these proxy measures do not directly capture clinical benefits at the individual-patient or societal level. Performance metrics that extend beyond technical accuracy such as mortality, adverse events or development of AMR would be more intuitive for clinicians and would better reflect whether AI models meaningfully improve quality of care.^65,105^ However, the lack of longitudinal patient follow-up across different levels of care often prevents such outcome-based evaluation.^108^

### Evaluation frameworks

A further limitation relates to model evaluation frameworks and performance reporting. Currently, reporting of CDSS outcomes is fragmented and inconsistent across studies.^28,108^ A structured, standardized and comprehensive approach to documenting CDSS model design, evaluation methods and results is essential to facilitate peer review, enable reproducibility and allow for robust assessment of model utility and potential biases. To promote transparency and comparability, studies should adhere to established reporting frameworks such as the Transparent Reporting of a Multivariable Prediction Model for Individual Prognosis or Diagnosis (TRIPOD), or the more recent ML–specific version, TRIPOD-ML.^105^

Model evaluation and validation is often limited to retrospective analyses using single-centre datasets, with relatively few prospective studies conducted in real-world clinical settings. Prospective studies are crucial in revealing the true clinical utility of these AI systems. Though limited in number, prospective studies (Table 3) have shown promising results on real-world clinical metrics such as length of stay, readmission probability, probability of therapy discontinuation and treatment success rates.^35,43,111^ These studies suggest that despite the current scarcity of prospective research, AI–driven interventions and predictive analytics hold promise for improving both patient outcomes and healthcare system efficiency related to infection management.

**Table 3.** Summary of prospective studies using AI-CDSS in ID

References	Decision support	AI methods	Prospective data	Outcome
Shimbukuro *et al*.^35^	Sepsis prediction	Gradient boosted trees	Single centre—142 patients	Length of stay and in-hospital mortality
Burdick *et al*.^43^	Sepsis prediction	Gradient boosted trees	75 147 patient encounters from early 2017 to mid-2018—multicentre	In-hospital mortality, length of stay and 30-day readmission rates
Shimbukuro *et al*.^88^	Derive novel-prescribing policies	Rule induction algorithm	515 prescriptions—single centre	Alert rate
Elligsen *et al*.^89^	Probability of antibiotic sensitivity	Logistic regression in addition to AMS pharmacist	201 patients—single centre	Antibiotic cessation and adequacy of treatment
Rawson *et al*.^17^	Infection prediction	SVM	140 patients—single centre	Alignment with prescription of treatment
Adams *et al*.^55^	Sepsis prediction/stratification	Mixture model of Cox proportional hazard models	590 736 patients—multicentre	Mortality rate, organ failure rate and length of stay
				
Herter *et al*.^111^	Outcome/treatment selection	Decision trees	2423 patients—multicentre	Proportion of successful treatments
Kallonen *et al*.^112^	Sepsis diagnosis	CNN	309 patients—multi centre	Performance and median early detection
Herman *et al*.^113^	Diagnosis of pulmonary rifampicin-resistant tuberculosis	ANN	157 participants—singe centre	Performance

Approaches range from sepsis prediction using gradient boosted trees to antibiotic sensitivity estimation via, logistic regression, with study sizes varying from single-site cohorts to multicentre datasets.

### Clinical integration and adoption

To be truly effective, AI-CDSS should provide augmented decision-making by combining computerized precision with clinician expertise. There is still a lack of consideration for prescriber engagement in the development and deployment of these tools.^108,114^ Ensuring alignment with clinical workflows, engaging end-users early in the design process and encouraging AI literacy among healthcare professionals are essential steps in fostering trust and adoption.^12,114^

Successful uptake also depends on local readiness, including local infrastructure and interoperability with existing EHR systems. Additionally, regular auditing and iterative refinement based on user feedback are essential for improving system usability, minimizing human error and ultimately improving quality of care.^11,91,115^

### Regulatory and ethical considerations

Developing moral and socially responsible AI–driven CDSS requires robust ethical and regulatory frameworks. In infection management and particularly in antibiotic prescribing, decision support tools must strike a balance between the needs of an individual patient and those of the wider society.^91^ At the practical level, key ethical concerns for the introduction of AI in ID extend beyond model performance to issues of transparency, fairness, accountability and governance.^91^

A central concern is algorithmic transparency. Clinicians must be able to understand and interpret algorithm recommendations to preserve clinical autonomy. However, many high-performing models operate as ‘black boxes’, making it difficult to trace how an algorithm reached a particular output.^116^ Research into explainable AI is widely ongoing and aims to address this limitation by improving the interpretability of complex models, enabling clinicians to critically evaluate CDSS reasoning.^117^ Ethical concerns also arise around data privacy and governance. Federated learning, where algorithms collaboratively learn by sharing model parameters without sharing data, may offer partial solutions but remains logistically challenging.^118^

Ensuring explainability, data privacy and shared accountability between developers and end-users is essential in building trustworthy, safe AI tools for clinical decision support. Importantly, robust ethical frameworks integrated into designing these systems will likely support their adoption into clinical practice.

### Future perspectives for AI-CDSS

A promising avenue to improve the development and implementation of AI-CDSS is the integration of decision support across the full sequence of treatment decisions. Many CDSS are currently designed to operate independently addressing a single clinical task. This fragmented approach limits their capacity to support holistic, longitudinal patient care. In infection prevention, for example, most studies focus narrowly on surveillance. The separation between infection prevention control (IPC) and antibiotic stewardship creates a disconnect, hindering a broader understanding of how prevention efforts impact downstream treatment decisions and antimicrobial use.

In contrast, adaptive treatment sequences offer a more comprehensive framework by adjusting therapeutic interventions over time based on the evolving health status of the patient while considering prior clinical decisions and their associated outcomes. Rather than addressing individual clinical decisions in isolation, adaptive treatment strategies recognize that therapeutic choices can influence, if not determine, the effectiveness of future interventions and vice versa. This adaptive, sequential approach enables individualized treatment adjustments that dynamically respond to a patient’s changing needs. As a result, clinical benefits can be balanced with potential risks while optimizing both short- and long-term outcomes.^119^

DT platforms have emerged as a promising technology with the potential to capture a patient’s evolving health status as a function of sequential clinical decisions. DTs are virtual replicas of complex physical systems, designed with various degrees of connectivity between virtual and physical counterparts. The standardized definition proposed by the National Academies of Science, Engineering, and Medicine (NASEM) states that a DT must be dynamically updated to mirror its physical twin and have predictive capabilities that can inform real-world decisions.^120^

The potential of DTs in healthcare has been increasingly recognized, as reflected by a growing number of publications exploring their application in medical decision-making and prediction.^121,122^ Notably, DTs of the human heart were shown to improve diagnosis, prognosis and treatment in cardiovascular diseases.^123,124^ A further example is the artificial pancreas, which functions as a closed-loop system to automate insulin management in Type I diabetes patients.^125–127^ This growing interest in DT for healthcare applications coincides with technological advances in the Internet of Things (IoT) and biosensor systems that enable the bidirectional, near-real-time flow of information between the physical and virtual counterparts.^128^

A more challenging task lies in modelling multi-organ systems and whole patients using DT approaches. Some examples of human DTs (HDTs) have been described in the literature, with a large proportion focusing on cardiology and metabolic diseases.^127,129–136^ The predominance of DT in these fields likely reflects the availability of abundant, high-frequency longitudinal data (ECGs, medical imaging and wearable glucose monitors) as well as well-defined, quantifiable clinical outcomes that facilitate model validation.

While fully autonomous DTs in cardiology have been developed to act directly on the patient through cardiac defibrillators,^133^ acting upon a human system without implantable devices remains difficult to achieve and requires a human-in-the-loop approach to implement clinical action. In this way, DTs can be conceptualized as a comprehensive, personalized and dynamic CDSS that continuously integrate medical data and simulate possible patient trajectories, enabling actionable intervention for precision care.^137–141^

The core principles of DTs, interoperability, modular architecture and scalability provide considerable advantages over traditional AI approaches to clinical modelling. Interoperability allows DTs to utilize data from multiple heterogenous sources, supporting a seamless flow of information that enables a holistic view of patient states. In infection management, advances in AI and multi-modal data fusion could allow for patient-level DTs that mirror the complexity of ID. Through the integration of heterogenous data including microbiology results (genomics and resistance phenotype), physiological parameters (biomarkers, vital signs and organ-function trends) and clinical interventions (diagnostics, medical history and treatment changes), the HDT could dynamically model the evolving interaction between pathogen, host and therapy.^121^ A HDT in infection management would allow for *in silico* simulations of clinical scenarios and forecasting of patient responses to clinical interventions.

The modular architecture of DTs also facilitates the integration of decision-making algorithms that currently operate in isolation while mirroring the multiple components of infection (pathogen, immune-response module and therapy) (Figure 2). This design allows DTs to be dynamically adapted to the evolving landscape of scientific knowledge and to the technological advances in medical data acquisition.^141^

At the practical level, the implementation of a human DT would likely benefit from a hybrid strategy combining mechanistic modelling with empirical, ML–driven approaches to build a reliable infrastructure where probabilistic simulations of clinical intervention outcomes can be run. An early example is described by Barbiero *et al.*,^142^ who developed a proof-of-concept DT framework using graph neural networks (GNNs). While the framework maintains a mechanistic representation of inter-organ relationships, the underlying dynamics are learned empirically from data to predict physiological parameters, thereby representing the evolution of the patient health state.

To further expand the potential of DTs for precision medicine, digital health technologies such as wearable devices and biosensors can be integrated with AI–driven DT models. Chemical biosensors can be used for real-time drug monitoring to account for inter-individual variability in antimicrobial pharmacokinetics related to comorbidities or concurrent medications, thereby ensuring antimicrobial efficacy and reduced toxicity while also mitigating the risk of AMR. Real-time therapeutic monitoring using biosensors to support antimicrobial therapeutic drug monitoring together with AI techniques such as RL could provide a promising strategy for precision drug delivery through closed-loop control and AI–driven treatment recommendations.^107^ Leveraging AI methodologies and real-time patient data to model the dynamic evolution of patient states would encourage decentralized healthcare and streamlined clinical management across different levels of care.

An important consideration is what constitutes ‘real-time’ in the clinical context. In most hospital settings, patient data such as vital signs and laboratory results are not continuously updated reflections of physiology but are instead produced at discrete intervals. However, when these parameters become available at the frequency required for bedside decision-making, they effectively represent real-time information within the clinical workflow. In other words, real-time should be defined relative to the timescale of clinical action rather than to continuous physiological monitoring. This distinction is crucial for DT frameworks in infection management, where decision support must align with how and when information is generated, interpreted and acted upon in the clinic.

A broader perspective on AI-CDSS is to integrate DT models into a network at the hospital or population level, enabling the modelling of patient–patient interactions to prevent outbreaks and unnecessary infections. Such an approach could reduce the burden on many of the strategies explored here, ultimately allowing for a more optimized allocation of resources and time. The necessary technologies and modelling techniques are available, making the development of a comprehensive prevention approaches feasible. The priority now is to demonstrate the practicality of enhanced infection control using such systems.

### Conclusions

AI-CDSS have the potential to improve bacterial infection management, contribute to reductions in AMR and enhance patient outcomes. Current tools remain limited in scope, offering rigid, narrowly defined guidance that falls short of the nuanced, adaptive support clinician desire. AI-CDSS implementation into clinical workflows is further hindered by a lack of prospective validation, minimal clinician engagement and insufficient evidence of real-world clinical impact.

To move beyond single-task models, future AI-CDSS should recognize the complexity of clinical care and provide dynamic, personalized decision support. This requires shifting from standalone algorithms towards integrated platforms that combine prevention, diagnosis and treatment adaptation into a cohesive environment.

Ultimately, AI tools should not replace clinical expertise but provide augmented decision-making by integrating computerized precision with clinical experience and intuition.^143^ When designed and implemented responsibly, AI-CDSS may serve as powerful tools in infection management to deliver better, safer and more equitable healthcare.
